# Real-world analysis of treatment patterns, effectiveness, and safety of daratumumab-based regimens in Chinese patients with newly diagnosed or relapsed/refractory multiple myeloma

**DOI:** 10.1186/s12885-025-13925-3

**Published:** 2025-05-07

**Authors:** Luqun Wang, Wei Yang, Yafei Wang, Ting Niu, Rong Fu, Yuping Zhong, Wenbin Qian, Kaiyang Ding, Kai Sun, Hong Liu, Baijun Fang, Hui Liu, Yanhui Li, Yishen Yang, Jianmin Zhuo, Xi Chen, Canchan Cui, Jin Lu

**Affiliations:** 1 Qilu Hospital of Shandong University, Shandong, China; 2 Shengjing Hospital of China Medical University, Liaoning, China; 3 Tianjin Medical University Cancer Institute and Hospital, National Clinical Research Center for Cancer, Tianjin’s Clinical Research Center for Cancer, Tianjin, China; 4 West China Hospital Sichuan University, Sichuan, China; 5 Tianjin Medical University General Hospital, Tianjin, China; 6 Qingdao Municipal Hospital, Qingdao, China; 7 Second Affiliated Hospital of Zhejiang University, Zhejiang, China; 8 First Affiliated Hospital of University of Science and Technology of China, Anhui, China; 9 Henan Provincial People’s Hospital, Henan, China; 10 Affiliated Hospital of Nantong University, Jiangsu, China; 11 Henan Cancer Hospital, Henan, China; 12 Beijing Hospital, Beijing, China; 13 Johnson & Johnson, Beijing, China; 14 IQVIA, Beijing, China; 15 Johnson & Johnson, Shanghai, China; 16 Peking University People’s Hospital, National Clinical Research Center for Hematologic Disease, No.11 Xizhimen South Street, XiCheng District, Beijing, 100044 People’s Republic of China; 17 Collaborative Innovation Center of Hematology, Soochow, China

**Keywords:** Daratumumab, Immunomodulatory drugs, Multiple myeloma, Proteasome inhibitors, Real-world evidence, Overall response rate, Baseline characteristics

## Abstract

**Background:**

Daratumumab is a human IgGκ monoclonal antibody targeting CD38 with direct on-tumor and immunomodulatory mechanisms of action. Daratumumab-based treatment is a standard of care for multiple myeloma (MM) based on data from randomized controlled trials. Real-world studies, such as that presented here from China, provide important data to complement randomized trials.

**Methods:**

This ongoing observational study describes real-world treatment patterns and outcomes among patients with symptomatic, newly diagnosed or relapsed/refractory MM treated with daratumumab in China. Patients must have received ≤ 3 prior lines of MM therapy. Data were collected prospectively and/or retrospectively, depending on time of treatment initiation. The primary study objective was to describe treatment patterns and clinical outcomes, and the secondary objective was to assess the safety and tolerability of daratumumab treatment.

**Results:**

As of the cutoff date (April 30, 2023) for this analysis, 212 patients had received ≥ 1 dose of daratumumab at 13 sites in China. Regimens included daratumumab monotherapy (*n* = 22) and daratumumab combined with dexamethasone only (*n* = 21), proteasome inhibitors (PIs) ± dexamethasone (*n* = 57), immunomodulatory drugs (IMiDs) ± dexamethasone (*n* = 72), PIs and IMiDs ± dexamethasone (*n* = 29), and other combinations (*n* = 11). Daratumumab was initiated by 16.5%, 53.3%, 16.5%, and 13.7% of patients across the first, second, third, and fourth lines of therapy, respectively. A best overall response of partial response or better was achieved by 71.8% of evaluable patients and very good partial response or better was achieved by 51.4% of patients. Estimated 6-month and 12-month progression-free survival rates were 84.3% and 75.0%, respectively. Outcomes were generally more favorable with daratumumab-based combinations than with daratumumab monotherapy. Serious treatment-emergent adverse events were reported in 13.7% of patients, with pneumonia (4.7%) the only serious event in ≥ 5 patients. The most frequently reported adverse drug reactions were leukopenia (6.6%), neutropenia (5.7%), and thrombocytopenia (5.7%).

**Conclusions:**

This observational study provides real-world insights into treatment decisions for Chinese patients with MM. The effectiveness and safety results support the use of daratumumab-based treatment as a standard-of-care therapy in Chinese patients with newly diagnosed or relapsed/refractory MM. This study is ongoing, with continued collection of outcomes data during a longer follow-up.

**Supplementary Information:**

The online version contains supplementary material available at 10.1186/s12885-025-13925-3.

## Introduction

Daratumumab is a human immunoglobulin G kappa (IgGκ) monoclonal antibody targeting CD38 with direct on-tumor [1–4] and immunomodulatory mechanisms of action [5–7]. Several randomized controlled trials (RCTs) have established daratumumab-based treatment as a standard of care globally for patients with newly diagnosed multiple myeloma (NDMM) or relapsed/refractory multiple myeloma (RRMM) [8–14], and they have provided pivotal evidence supporting its approval in multiple countries globally. Daratumumab was first approved in China in 2019 and is currently indicated in combination with lenalidomide/dexamethasone (Rd) or bortezomib/melphalan/prednisone (VMP) for patients with NDMM who are ineligible for autologous stem cell transplant, as monotherapy for patients with RRMM whose prior therapy included a proteasome inhibitor (PI) and immunomodulatory drug (IMiD), and in combination with Rd or bortezomib/dexamethasone (Vd) in patients with RRMM who received ≥ 1 prior therapy. Clinical trials in Chinese patients have shown daratumumab to be well tolerated and efficacious, both in patients with NDMM and in those with RRMM [15–17].

Despite the importance of RCTs in evaluating new therapies, they do not always reflect routine clinical practice, and many patients with MM are excluded from clinical trials. Real-world studies, on the other hand, play a vital role in gathering broader data regarding patient and disease characteristics, treatment decisions, and overall patient management. This allows for patient profiles to be distinguished and for greater insights into how certain patients and disease characteristics may impact treatment decisions in clinical practice. Furthermore, real-world studies help identify common treatment patterns and utilization, providing additional insights into routine clinical practice. Thus, real-world studies provide data that complement clinical trial data, allowing for greater generalization to the general population.

Here, we report results from the first interim analysis of a multicenter, observational registry study that describes the real-world treatment patterns and outcomes in routine clinical practice among patients with multiple myeloma (MM) who were treated with daratumumab in China. This is one of the first and largest studies to enroll a substantial population of Chinese patients with MM who were treated with daratumumab in the real-world setting, with data collected from a total of 13 clinical practices across China.

## Methods

### Study design and patients

This ongoing, multicenter, noninterventional, observational registry study (Chinese Clinical Trial Registry Identifier: ChiCTR2200055491; registered January 10, 2022) is enrolling Chinese patients who have been diagnosed with symptomatic NDMM or RRMM. The study was conducted in accordance with the ethical principles outlined in the Declaration of Helsinki, Good Clinical Practices, and abided by all applicable regulatory requirements. The study protocol and amendments were reviewed and approved by the Independent Ethics Committees or Institutional Review Boards at each participating site. Each patient provided written informed consent prior to data collection.

All patients were adults aged ≥ 18 years and belonged to 1 of 2 groups: (1) they started daratumumab treatment after August 1, 2019, and were expected to continue ongoing daratumumab at the time of study initiation (November 3, 2021); or (2) they started daratumumab treatment after study initiation. The decision to treat with daratumumab must have been made prior to and independently of the patient’s inclusion in the study, and treatment was administered in accordance with local clinical practice and physician discretion. Patients were excluded from the study if they had received ≥ 4 prior lines of MM therapy before starting daratumumab-based treatment or had a diagnosis of other cancers before their MM diagnosis (excluding squamous and basal cell skin carcinoma or carcinoma in situ of the cervix or breast considered to be cured).

Data were collected prospectively for patients who started daratumumab after study initiation. For patients who started daratumumab after August 1, 2019, but before study initiation, data were collected both retrospectively through medical chart review and prospectively after enrollment. Study baseline was defined as the latest status before the first dose of daratumumab.

Enrollment was planned for 1 year, followed by a 2-year follow-up period after the last patient was enrolled. Patients were discontinued from the study (with no further data collection) if any of the following reasons were met: lost to follow-up, withdrawal of consent, death, or other reasons (e.g., physician’s decision, administrative reasons).

Two interim analyses of the study data (at 4 and 12 months after the last patient was enrolled) are planned to be performed separately, with a purpose of reporting the clinical outcomes but without the intention for a decision to stop or continue the study. The first interim analysis, described here, was performed using data with a cut-off date of April 30, 2023.

### Study endpoints

The primary study objective is to describe treatment patterns and clinical outcomes in routine clinical practice among patients with MM treated with daratumumab in China, and the secondary objective is to assess the safety and tolerability of daratumumab treatment. The intended frequency for data collection was every 2 months for the first 12 months after patient enrollment and thereafter every 6 months until study end, defined as 2 years after the last patient enrollment or the last dosing patient with progressive disease or death, whichever comes first. The overall study duration is expected to be approximately 3 years. Data collected at baseline and/or during the observation period encompassed patient demographics, medical history, comorbidities, disease characteristics, previous therapies, current daratumumab therapies, subsequent non-daratumumab therapies, treatment effectiveness, and safety.

Treatment response outcomes were evaluated in accordance with the International Myeloma Working Group (IMWG) response criteria. This first interim analysis included overall response rate (ORR), progression-free survival (PFS), and overall survival (OS). Safety outcomes, such as serious treatment-emergent adverse events (TEAEs) and adverse drug reactions, were collected throughout the daratumumab treatment period from retrospective and/or prospective periods under a real-world setting.

Baseline demographic and clinical characteristics, treatment patterns, clinical outcomes, and safety outcomes were analyzed overall and by patient subgroups. Daratumumab-based regimen subgroups were categorized as follows: daratumumab monotherapy, daratumumab in combination with dexamethasone, daratumumab in combination with a PI (bortezomib, carfizomib, and ixazomib) ± dexamethasone, daratumumab in combination with an IMiD (thalidomide, lenalidomide, and pomalidomide) ± dexamethasone, daratumumab in combination with a PI and IMiD ± dexamethasone, and daratumumab in any other regimen not listed.

### Statistics

No formal sample size calculation was performed. Sample size was determined pragmatically based on the typical number of patients with MM treated with daratumumab-based regimens or other regimens at participating sites and on cost. The expected sample size was approximately 220 patients.

In this first interim analysis, clinical outcomes were analyzed during the daratumumab-based treatment period, defined as the time between initiation of daratumumab and commencement of next non-daratumumab therapy line. Considering the potentially various types and sparse frequency of visits in a real-world setting, best response was defined as the true best response among all responses collected prior to reported progressive disease. The ORR was the proportion of patients who had a best response of complete response (CR), very good partial response (VGPR), or partial response (PR) during daratumumab-based treatment. PFS was defined as the length of time from the daratumumab initiation date (date of first daratumumab dose) until first documented disease progression or death due to any cause. If progression data were not collected during the daratumumab-based treatment period but a patient initiated a subsequent line of non-daratumumab therapy, the start date of the subsequent therapy line was used as a proxy for the PFS event date.

Continuous and categorical variables were summarized using descriptive statistics, and the Kaplan-Meier method was used to estimate time-to-event variables.

The ORR was based on the response-evaluable population, which included all patients who received ≥ 1 dose of daratumumab and had ≥ 1 tumor response evaluation, disease progression, or death. PFS and safety analyses were based on the safety population, which included all enrolled patients who received ≥ 1 dose of daratumumab.

## Results

### Patient characteristics

The first patient’s first dose of daratumumab was received on September 15, 2019, with data collected retrospectively from medical chart review. As of the cutoff date (April 30, 2023) for this first interim analysis, 212 patients who received ≥ 1 dose of daratumumab have been enrolled in the study from 13 participating sites in China and followed for a median (interquartile range) of 10.5 (7.2–15.3) months. The majority (79.2%) of patients had initiated daratumumab prior to study initiation (November 3, 2021), with at least a portion of their data collected retrospectively. Patient characteristics are presented in Table 1. Median (range) age at the time of MM diagnosis was 61 (29–89) years and at study baseline was 64 (29–89) years. Most patients had an Eastern Cooperative Oncology Group (ECOG) performance status of 0 or 1 (82.4%). A substantial proportion of patients had International Staging System (ISS) stage III disease (41.1%) and/or Revised ISS stage III disease (33.8%). Renal insufficiency/failure was indicated by the investigator for 32 (15.1%) patients. A total of 76 patients were evaluable for cytogenetic risk, among which 38 (50.0%) were considered to have high-risk cytogenetics, defined as the presence of ≥ 1 of the following cytogenetic abnormalities at baseline: t(4:14) (*n* = 10 [13.2%]), t(14:16) (*n* = 3 [3.9%]), del(17p) (*n* = 8 [10.5%]), gain(1q21) (*n* = 23 [30.3%]), amp(1q21) (*n* = 7 [9.2%]), and/or t(14:20) (*n* = 2 [2.6%]). A total of 114 patients had hepatitis B virus (HBV) test results at baseline; 7 of 112 (6.3%) patients tested positive for HBV surface antigen and 28 of 112 (25.0%) patients tested positive for HBV core antibodies at baseline (Additional file 1, Table A).

**Table 1:** Patient demographics and clinical characteristics in the safety population

	Overall (*n *= 212)	Daratumumab monotherapy (*n *= 22)	Daratumumab + dexamethasone (*n *= 21)	Daratumumab + PI ± dexamethasone (*n *= 57)	Daratumumab + IMiD ± dexamethasone (*n *= 72)	Daratumumab + PI + IMiD ± dexamethasone (*n *= 29)	Daratumumab + other agents (*n *= 11)
Age at baseline, y							
Mean (SD)	62.7 (9.7)	65.8 (10.2)	63.0 (10.3)	62.6 (9.8)	63.2 (9.2)	59.5 (9.6)	61.1 (10.2)
Median (range)	64 (29–89)	66 (46–81)	64 (42–83)	63 (37–83)	64.5 (29–89)	59 (38–76)	67 (46–75)
Sex, *n* (%)							
Male	122 (57.5)	12 (54.5)	10 (47.6)	40 (70.2)	37 (51.4)	15 (51.7)	8 (72.7)
Female	90 (42.5)	10 (45.5)	11 (52.4)	17 (29.8)	35 (48.6)	14 (48.3)	3 (27.3)
Time from diagnosis to daratumumab initiation, y							
*n*	211	22	21	57	71	29	11
Mean (SD)	1.9 (2.5)	2.6 (2.4)	2.8 (3.1)	1.3 (1.8)	2.2 (2.6)	1.2 (2.6)	2.4 (2.2)
Median (range)	1 (0–12)	2 (0–8)	1 (0–9)	0 (0–6)	2 (0–12)	0 (0–12)	1 (0–7)
MM isotype at initial diagnosis, *n* (%)							
*n*	207	21	19	57	70	29	11
IgG	104 (50.2)	7 (33.3)	10 (52.6)	25 (43.9)	39 (55.7)	17 (58.6)	6 (54.5)
IgA	37 (17.9)	5 (23.8)	3 (15.8)	11 (19.3)	14 (20.0)	3 (10.3)	1 (9.1)
IgD	13 (6.3)	2 (9.5)	2 (10.5)	4 (7.0)	1 (1.4)	2 (6.9)	2 (18.2)
IgM	1 (0.5)	0	0	0	1 (1.4)	0	0
Light chain	46 (22.2)	7 (33.3)	3 (15.8)	13 (22.8)	14 (20.0)	7 (24.1)	2 (18.2)
Biclonal	0	0	0	0	0	0	0
Nonsecretory	1 (0.5)	0	0	1 (1.8)	0	0	0
Other	5 (2.4)	0	1 (5.3)	3 (5.3)	1 (1.4)	0	0
MM stage at diagnosis, *n* (%)							
By ISS							
*n*	141	14	15	33	53	21	5
I	30 (21.3)	2 (14.3)	4 (26.7)	3 (9.1)	15 (28.3)	6 (28.6)	0
II	53 (37.6)	7 (50.0)	7 (46.7)	8 (24.2)	20 (37.7)	10 (47.6)	1 (20.0)
III	58 (41.1)	5 (35.7)	4 (26.7)	22 (66.7)	18 (34.0)	5 (23.8)	4 (80.0)
By R-ISS							
*n*	68	6	5	18	28	6	5
I	9 (13.2)	1 (16.7)	2 (40.0)	1 (5.6)	3 (10.7)	1 (16.7)	1 (20.0)
II	36 (52.9)	4 (66.7)	3 (60.0)	6 (33.3)	17 (60.7)	4 (66.7)	2 (40.0)
III	23 (33.8)	1 (16.7)	0	11 (61.1)	8 (28.6)	1 (16.7)	2 (40.0)
ECOG performance status, *n* (%)							
*n*	131	18	12	31	44	20	6
0	31 (23.7)	4 (22.2)	3 (25.0)	8 (25.8)	12 (27.3)	3 (15.0)	1 (16.7)
1	77 (58.8)	9 (50.0)	7 (58.3)	19 (61.3)	23 (52.3)	15 (75.0)	4 (66.7)
2	15 (11.5)	4 (22.2)	1 (8.3)	3 (9.7)	4 (9.1)	2 (10.0)	1 (16.7)
≥ 3	8 (6.1)	1 (5.6)	1 (8.3)	1 (3.2)	5 (11.4)	0	0

*ECOG *Eastern Cooperative Oncology Group, *Ig *Immunoglobulin, *IMiD *Immunomodulatory drug, *ISS *International staging system, *MM *Multiple myeloma, *PI *Proteasome inhibitor, *R-ISS *Revised international staging system, *SD *Standard deviation

### Treatment patterns

**Fig. 1:**
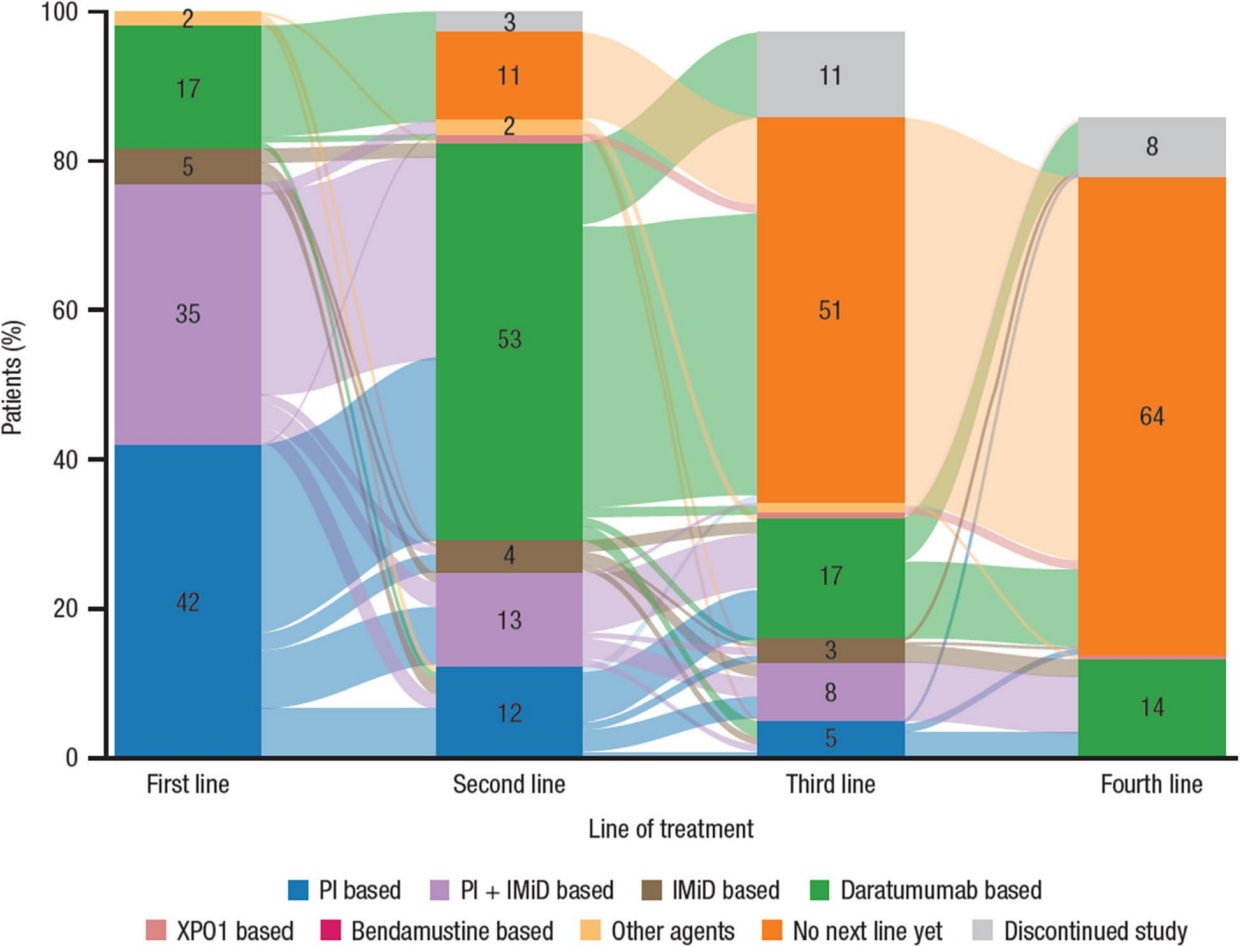
Sankey diagram of regimen use by therapy line in the safety population. Percentages for each line are calculated based on a denominator of 212 patients. The bar for each therapy line represents patients at risk for that specific line. The bar for third-line therapy does not total 100% because 3% of patients discontinued second-line treatment; the bar for fourth-line therapy does not total 100% because 14% of patients cumulatively had discontinued the study. IMiD, immunomodulatory drug; PI, proteasome inhibitor

As illustrated in Fig. 1, in the safety population, PI-based and PI + IMiD–based regimens were primarily used as first-line treatment (42% and 35% of patients, respectively), and the majority of patients who received these regimens in the first-line setting transitioned to daratumumab-based therapy in the second-line setting. Although only 17% of patients received daratumumab-based regimens in the first-line setting, daratumumab-based regimens were predominantly used as second-line therapy (53% of patients), and the majority of patients who began a daratumumab-based regimen in the second-line setting had transitioned from previously receiving PI-based or PI + IMiD–based regimens. Approximately half (51%) of the patients had not yet received third-line treatment.

A summary of previous non-daratumumab therapies is provided in Table 2. Most patients (83.5%) had received ≥ 1 prior line of therapy before initiating daratumumab, with about half (53.3%) of patients having received only 1 prior line of therapy. PIs were widely used by Chinese patients, with 82.1% of patients previously exposed to a PI, the most common being bortezomib (80.2%). However, the use of an IMiD was lower, with 58.0% of patients previously exposed to an IMiD, the most common being lenalidomide (51.9%). Of the 121 patients who received combination regimens prior to initiating daratumumab, the most predominant combinations were bortezomib/lenalidomide/dexamethasone (VRd; 38.2%), Vd (12.3%), and Rd (9.9%).

**Table 2:** Prior non-daratumumab treatment patterns for multiple myeloma

*n* (%)	Overall (*n *= 212)	Daratumumab monotherapy (*n *= 22)	Daratumumab + dexamethasone (*n *= 21)	Daratumumab + PI ± dexamethasone (*n *= 57)	Daratumumab + IMiD ± dexamethasone (*n *= 72)	Daratumumab + PI + IMiD ± dexamethasone (*n *= 29)	Daratumumab + other agents (*n *= 11)
Number of previous non-daratumumab MM therapy lines							
0	35 (16.5)	3 (13.6)	3 (14.3)	14 (24.6)	7 (9.7)	8 (27.6)	0
1	113 (53.3)	6 (27.3)	12 (57.1)	29 (50.9)	45 (62.5)	14 (48.3)	7 (63.6)
2	35 (16.5)	6 (27.3)	5 (23.8)	8 (14.0)	11 (15.3)	4 (13.8)	1 (9.1)
3	29 (13.7)	7 (31.8)	1 (4.8)	6 (10.5)	9 (12.5)	3 (10.3)	3 (27.3)
Previous non-daratumumab MM therapies							
IMiDs	123 (58.0)	16 (72.7)	10 (47.6)	22 (38.6)	52 (72.2)	15 (51.7)	8 (72.7)
Lenalidomide	110 (51.9)	13 (59.1)	10 (47.6)	19 (33.3)	48 (66.7)	13 (44.8)	7 (63.6)
Pomalidomide	17 (8.0)	5 (22.7)	1 (4.8)	4 (7.0)	3 (4.2)	2 (6.9)	2 (18.2)
Thalidomide	25 (11.8)	4 (18.2)	4 (19.0)	5 (8.8)	9 (12.5)	3 (10.3)	0
PIs	174 (82.1)	19 (86.4)	18 (85.7)	42 (73.7)	63 (87.5)	21 (72.4)	11 (100.0)
Bortezomib	170 (80.2)	18 (81.8)	17 (81.0)	42 (73.7)	61 (84.7)	21 (72.4)	11 (100.0)
Ixazomib	42 (19.8)	10 (45.5)	4 (19.0)	7 (12.3)	16 (22.2)	4 (13.8)	1 (9.1)

*IMiD *Immunomodulatory drug, *MM *Multiple myeloma, *PI *Proteasome inhibitor

The most frequent daratumumab-based therapies used by Chinese patients included daratumumab + IMiD ± dexamethasone (*n* = 72 [34.0%]; most common regimens being daratumumab/pomalidomide/dexamethasone [D-Pd; *n* = 40/72 (55.6%)] and daratumumab/lenalidomide/dexamethasone [D-Rd; *n* = 34/72 (47.2%)]) or daratumumab + PI ± dexamethasone (*n* = 57 [26.9%]; most common regimen being daratumumab/bortezomib/dexamethasone [D-Vd; *n* = 46/57 (80.7%)]). Additional therapies included daratumumab + PI + IMiD ± dexamethasone (*n* = 29 [13.7%]; most common regimen being daratumumab/bortezomib/lenalidomide/dexamethasone [D-VRd; *n* = 14/29 (48.3%)]), daratumumab monotherapy (*n* = 22 [10.4%]), daratumumab + dexamethasone only (*n* = 21 [9.9%]), and daratumumab + other agents (*n* = 11 [5.2%]; most common regimen was daratumumab/venetoclax/dexamethasone [VenDd; *n* = 4/11 (36.4%)]). The median (range) time from MM diagnosis to daratumumab initiation was 1 (0–12) year (Table 3). Most (62.3%) patients initiated daratumumab therapy because of a physician recommendation, the most common reason given by physicians being the deeper clinical responses observed in the real-world with daratumumab. In particular, physician recommendation was a key reason for the initiation of daratumumab + PI + IMiD ± dexamethasone (75.9%) and daratumumab + PI ± dexamethasone (75.4%). Although baseline characteristics were generally comparable across the daratumumab-based therapy subgroups, patients who received daratumumab + PI + IMiD ± dexamethasone tended to be slightly younger and those who received daratumumab + PI ± dexamethasone or daratumumab + other agents were more likely to have ISS or Revised ISS stage III disease (Table 1).

**Table 3:** Daratumumab treatment patterns

	Overall (*n* = 212)	Daratumumab monotherapy (*n* = 22)	Daratumumab + dexamethasone (*n* = 21)	Daratumumab + PI ± dexamethasone (*n* = 57)	Daratumumab + IMiD ± dexamethasone (*n* = 72)	Daratumumab + PI + IMiD ± dexamethasone (*n* = 29)	Daratumumab + other agents (*n* = 11)
Reason for initiation of daratumumab therapy, *n* (%)							
*n*	209	21	21	56	71	29	11
Adverse event	10 (4.7)	0	1 (4.8)	5 (8.8)	3 (4.2)	1 (3.4)	0
Disease progression	52 (24.5)	9 (40.9)	4 (19.0)	8 (14.0)	23 (31.9)	4 (13.8)	4 (36.4)
Physician recommendation	132 (62.3)	8 (36.4)	11 (52.4)	43 (75.4)	41 (56.9)	22 (75.9)	7 (63.6)
Patient request	3 (1.4)	0	0	0	2 (2.8)	1 (3.4)	0
Unknown	20 (9.4)	4 (18.2)	5 (23.8)	3 (5.3)	6 (8.3)	2 (6.9)	0
Line of therapy at daratumumab initiation, *n* (%)							
First	35 (16.5)	3 (13.6)	3 (14.3)	14 (24.6)	7 (9.7)	8 (27.6)	0
Second	113 (53.3)	6 (27.3)	12 (57.1)	29 (50.9)	45 (62.5)	14 (48.3)	7 (63.6)
Third	35 (16.5)	6 (27.3)	5 (23.8)	8 (14.0)	11 (15.3)	4 (13.8)	1 (9.1)
Fourth	29 (13.7)	7 (31.8)	1 (4.8)	6 (10.5)	9 (12.5)	3 (10.3)	3 (27.3)
Number of daratumumab cycles							
Mean (SD)	5.6 (4.2)	5.9 (4.5)	5.1 (4.9)	5.5 (4.1)	6.1 (4.2)	4.5 (2.8)	5.5 (5.5)
Median (range)	5 (1–20)	5 (1–19)	3 (1–19)	5 (1–19)	5 (1–20)	4 (1–11)	3 (1–17)
Total duration of daratumumab exposure, mo							
Mean (SD)	8.3 (7.1)	8.9 (9.4)	7.5 (7.9)	8.2 (7.0)	8.5 (6.0)	8.0 (7.6)	7.7 (6.9)
Median (range)	6.5 (0–43.1)	7.2 (0.1–43.1)	4.9 (0.1–31.8)	6.5 (0.3–26.5)	7.4 (0–32.3)	4.2 (0.3–27.0)	8.4 (0.3–22.5)
Duration of daratumumab exposure in first line, mo							
*n*	35	3	3	14	7	8	0
Mean (SD)	10.9 (7.8)	9.7 (11.1)	9.5 (11.4)	12.4 (6.7)	12.6 (10.8)	7.9 (4.7)	-
Median (range)	9.7 (0.3–32.3)	7.0 (0.3–22.0)	5.1 (0.9–22.4)	9.8 (2.1–22.2)	10.5 (0.3–32.3)	7.6 (1.1–14.7)	-
Duration of daratumumab exposure in second line, mo							
*n*	113	6	12	29	45	14	7
Mean (SD)	7.8 (6.8)	13.3 (15.2)	8.7 (8.7)	6.1 (5.3)	8.3 (4.6)	6.8 (7.9)	6.8 (7.8)
Median (range)	6.2 (0–43.1)	8.5 (0.5–43.1)	5.6 (0.3–31.8)	5.3 (0.3–26.5)	7.6 (0–19.6)	3.9 (0.3–23.1)	5.0 (0.3–22.5)
Duration of daratumumab exposure in third line, mo							
*n*	35	6	5	8	11	4	1
Mean (SD)	8.3 (7.5)	8.2 (5.3)	4.1 (3.6)	9.6 (9.3)	7.8 (6.3)	14.6 (11.4)	0.8 (-)
Median (range)	5.8 (0.1–27.0)	9.3 (0.1–13.6)	3.3 (0.1–9.9)	6.6 (0.3–25.5)	6.9 (1.2–21.5)	13.7 (4.1–27.0)	0.8 (0.8–0.8)
Duration of daratumumab exposure in fourth line, mo							
*n*	29	7	1	6	9	3	3
Mean (SD)	6.9 (6.0)	5.4 (4.2)	4.7 (-)	6.9 (8.3)	7.4 (7.3)	4.7 (2.5)	12.0 (2.1)
Median (range)	4.7 (0.5–22.7)	4.4 (0.6–12.1)	4.7 (4.7–4.7)	2.9 (0.5–20.0)	4.7 (0.8–22.7)	3.4 (3.2–7.6)	11.6 (10.1–14.3)

*IMiD *Immunomodulatory drug, *PI *Proteasome inhibitor, *SD *Standard deviation

Median (range) daratumumab exposure time was 6.5 (0–43.1) months for the overall population. For regimen types with the greatest sample size, daratumumab + IMiD ± dexamethasone and daratumumab + PI ± dexamethasone, median (range) daratumumab exposure time was 7.4 (0–32.3) months and 6.5 (0.3–26.5) months, respectively (Table 3). Duration of daratumumab exposure was longer in earlier lines of therapy, with median (range) durations of 9.7 (0.3–32.3), 6.2 (0–43.1), 5.9 (0.1–27.0), and 4.7 (0.5–22.7) months when daratumumab was initiated in the first, second, third, and fourth lines, respectively (Additional file 2, Figure A). The mean number of daratumumab cycles received was 5.6 overall and varied between 4.5 cycles for patients receiving daratumumab + PI + IMiD ± dexamethasone and 6.1 cycles for those receiving daratumumab + IMiD ± dexamethasone. Overall, 100%, 86.8%, 75.0%, 61.3%, 50.9%, 15.6%, and 4.2% of patients were treated with at least 1, 2, 3, 4, 5, 10, and 15 cycles of daratumumab-based therapy, respectively. A total of 61 (28.8%) patients discontinued the trial; reasons were withdrawal by the patient (*n* = 27), death (*n* = 25; described below), lost to follow-up (*n* = 1), and other reasons (*n* = 8).

Among patients with renal insufficiency/failure (*n* = 32) as reported by their investigator, 11 (34.4%) received daratumumab + PI ± dexamethasone, while 8 (25.0%) received daratumumab + IMiD ± dexamethasone, 7 (21.9%) received daratumumab + dexamethasone, and 6 (18.8%) received daratumumab + PI + IMiD ± dexamethasone. The median daratumumab exposure time was 5.2 months.

As most patients were aged < 75 years (*n* = 191), daratumumab treatment patterns for this subgroup were consistent with the overall population. Among patients aged ≥ 75 years (*n* = 21), 8 (38.1%) patients received daratumumab + PI ± dexamethasone, 5 (23.8%) received daratumumab monotherapy, and 4 (19.0%) received daratumumab + IMiD ± dexamethasone. The median daratumumab exposure time among older patients was 9.7 months.

At the time of this analysis, 22 (10.4%) patients had undergone autologous stem cell transplant either during the period of daratumumab-based treatment or after discontinuing daratumumab-based treatment but before initiating a subsequent line of therapy.

### Treatment responses

Responses to daratumumab treatment overall and by daratumumab-based regimen are presented in Table 4 and Additional file 3, Figure B. Among the 181 (85.4%) patients in the response-evaluable population, the best ORR (PR or better) was 71.8% (*n* = 130). For the 2 most common daratumumab-based regimens, daratumumab + IMiD ± dexamethasone and daratumumab + PI ± dexamethasone, the ORRs were 75.0% and 71.1%, respectively. A best response of very good partial response or better (≥ VGPR) was achieved by 93 (51.4%) patients, with rates 49% or higher across all daratumumab-based regimens except daratumumab monotherapy (29.4%). A best response of complete response or better (≥ CR) was achieved by 55 (30.4%) patients; rates were 37.5% and 24.4% in the daratumumab + IMiD ± dexamethasone and daratumumab + PI ± dexamethasone regimens, respectively. The ORR remained high among patients who received daratumumab-based triplet regimens overall (73.4%), including those who received daratumumab + bortezomib ± dexamethasone (73.7%; Additional file 4, Table B). Responses to daratumumab by line of treatment are presented in Additional file 5, Table C. Response rates were higher when daratumumab was received in earlier treatment lines, at 79.3% to 80.4% for ORR and 61.8% to 62.1% for ≥ VGPR rates in the first/second-line settings compared with 48.1% to 52.2% for ORR and 22.2% to 26.1% for ≥ VGPR rates in the third/fourth-line settings.

**Table 4:** Response with daratumumab-based treatment

*n* (%)	Overall (*n* = 181)	Daratumumab monotherapy (*n* = 17)	Daratumumab + dexamethasone (*n* = 18)	Daratumumab + PI ± dexamethasone (*n* = 45)	Daratumumab + IMiD ± dexamethasone (*n* = 64)	Daratumumab + PI + IMiD ± dexamethasone (*n* = 27)	Daratumumab + other agents (*n* = 10)
ORR (≥ PR)	130 (71.8)	10 (58.8)	13 (72.2)	32 (71.1)	48 (75.0)	19 (70.4)	8 (80.0)
≥ VGPR	93 (51.4)	5 (29.4)	9 (50.0)	22 (48.9)	35 (54.7)	16 (59.3)	6 (60.0)
Response category							
Stringent CR	5 (2.8)	0	1 (5.6)	0	0	3 (11.1)	1 (10.0)
CR	50 (27.6)	2 (11.8)	6 (33.3)	11 (24.4)	24 (37.5)	4 (14.8)	3 (30.0)
VGPR	38 (21.0)	3 (17.6)	2 (11.1)	11 (24.4)	11 (17.2)	9 (33.3)	2 (20.0)
PR	37 (20.4)	5 (29.4)	4 (22.2)	10 (22.2)	13 (20.3)	3 (11.1)	2 (20.0)
Minimal response	4 (2.2)	2 (11.8)	0	1 (2.2)	1 (1.6)	0	0
Stable disease	18 (9.9)	2 (11.8)	1 (5.6)	5 (11.1)	6 (9.4)	4 (14.8)	0
Progressive disease	13 (7.2)	0	1 (5.6)	4 (8.9)	6 (9.4)	0	2 (20.0)
Clinical relapse	1 (0.6)	0	0	1 (2.2)	0	0	0
Not evaluable	14 (7.7)	3 (17.6)	2 (11.1)	2 (4.4)	3 (4.7)	4 (14.8)	0

*CR *Complete response, *IMiD *Immunomodulatory drug, *ORR *Overall response rate, *PI *Proteasome inhibitor, *PR *Partial response, *VGPR *Very good partial response

Analyses of response to daratumumab among patient subgroups of interest are presented in Additional file 4, Table B. The ORR remained high among patients with renal insufficiency/failure (78.6%), and irrespective of baseline cytogenetic risk status (standard risk, 78.1%; high risk, 82.9%); however, the ORR was numerically lower among patients aged ≥ 75 years (57.9%) than among younger patients (73.5%). The ORR ranged from 65.4% to 75.7% when evaluated by type of last prior line of therapy before daratumumab initiation. The ORR was also high (86.4%) among patients who underwent transplant during the period of daratumumab-based treatment or immediately afterward, before beginning a subsequent line of therapy; however, further follow-up will be required to permit additional data collection for a more robust analysis.

### Progression-free and overall survival

**Fig. 2:**
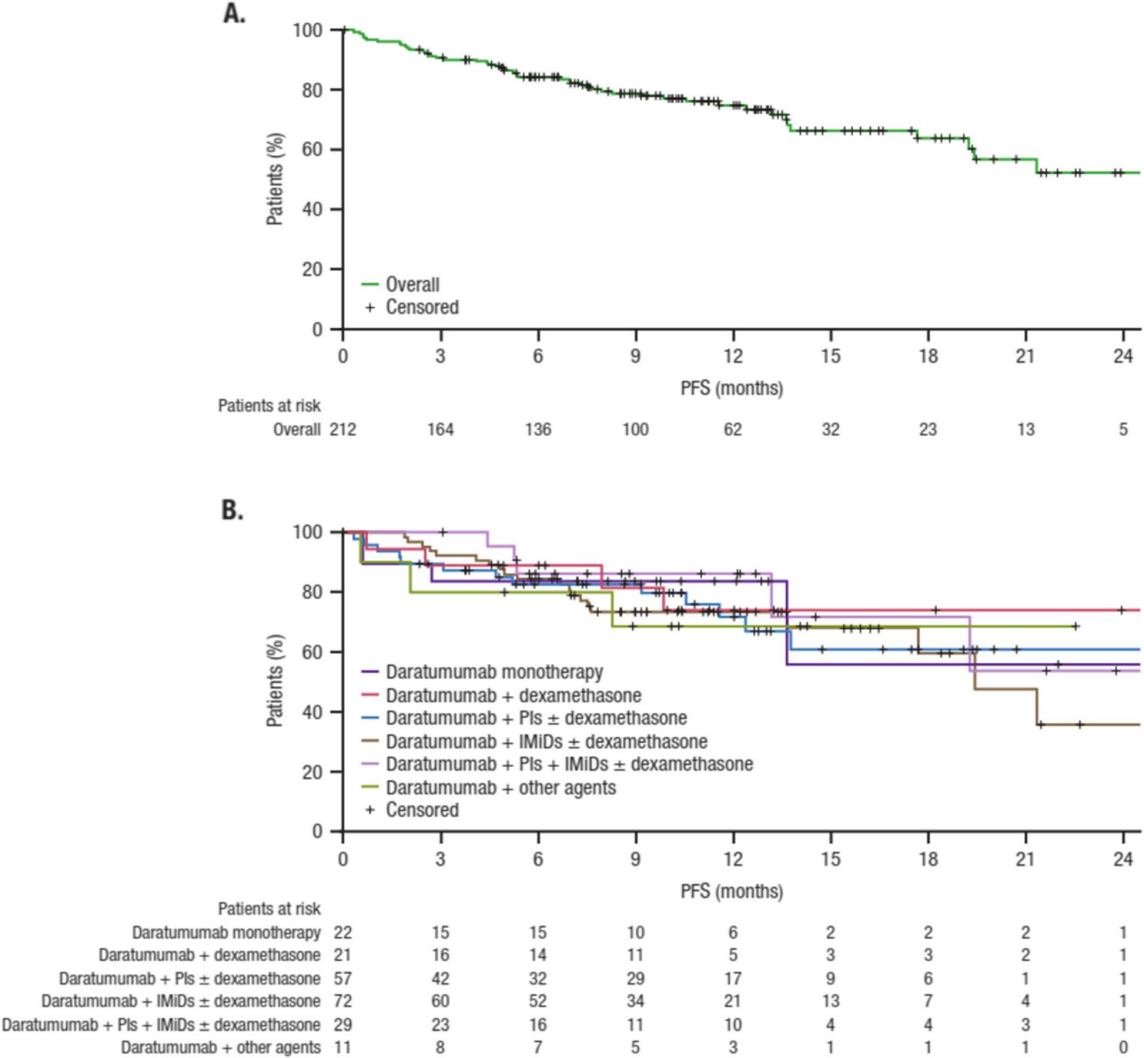
PFS overall (**A**) and by daratumumab-based regimen (**B**). IMiD, immunomodulatory drug; PFS, progression-free survival; PI, proteasome inhibitor

Results for survival parameters (PFS and OS) overall and by daratumumab-based regimen are presented in Additional file 6, Table D. Figure 2 presents Kaplan–Meier plots for PFS in the overall group (safety population) and by daratumumab-based regimen. At the time of this analysis, 49 (23.1%) patients experienced disease progression or death, and median PFS was not evaluable. The estimated 6-month PFS rate was 84.3%, with similar rates across the daratumumab-based regimens (range, 80.0%−88.9%), and the estimated 12-month PFS rate was 75.0%.

**Fig. 3:**
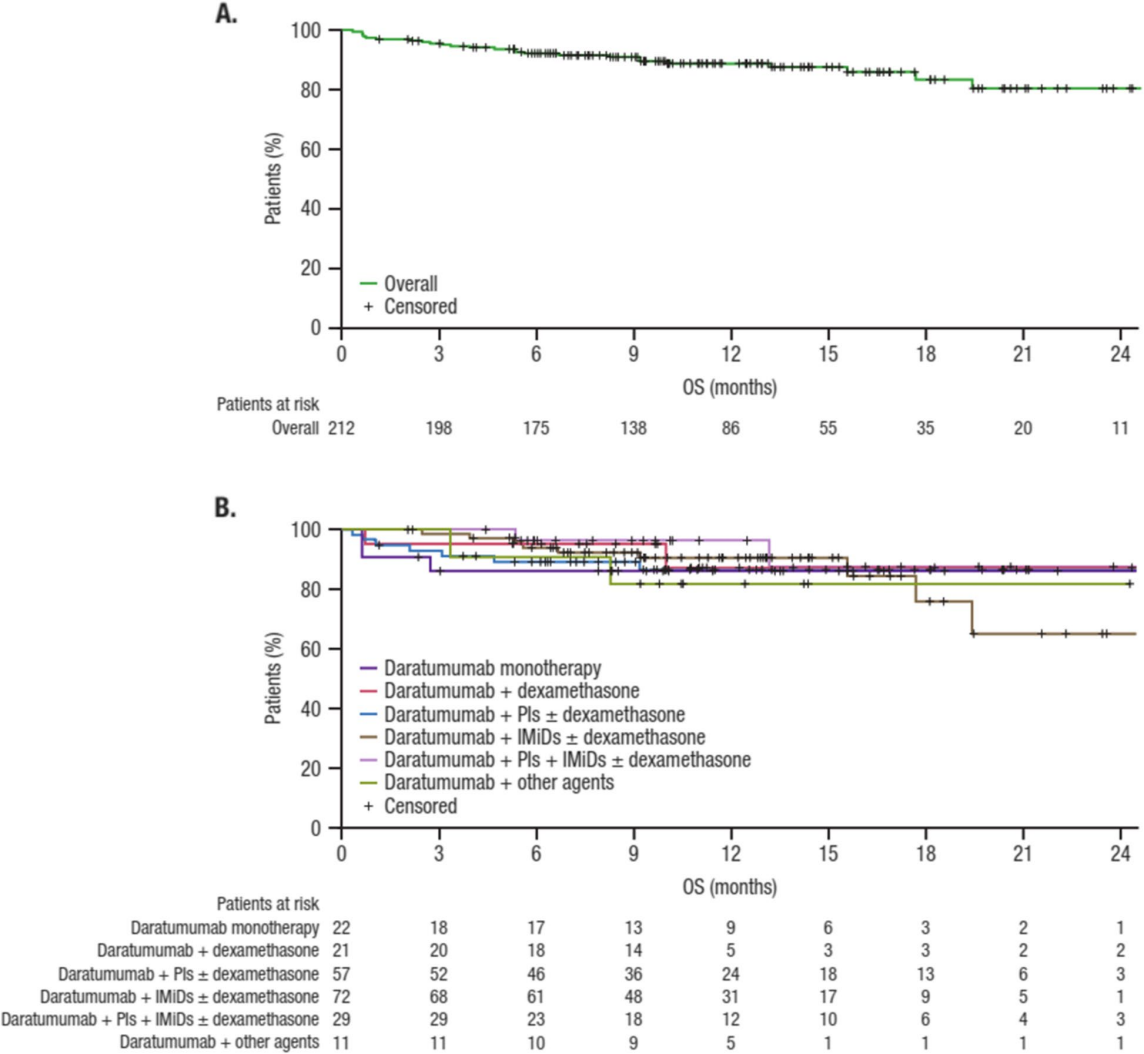
OS overall (**A**) and by daratumumab-based regimen (**B**). IMiD, immunomodulatory drug; OS, overall survival; PI, proteasome inhibitor

Figure 3 presents Kaplan–Meier plots for OS in the overall group (safety population) and by daratumumab-based regimen. Median OS was not evaluable, with estimated survival rates of 92.2% at 6 months and 88.9% at 12 months; estimated 12-month survival rates remained > 80% across daratumumab-based regimens. Survival rates at 6 months and 12 months decreased slightly with the initiation of daratumumab in later treatment lines, from 97.1% for both time points in the first-line setting to 95.5% and 91.7% in the second-line setting, 87.8% and 80.0% in the third-line setting, and 78.4% for both time points in the fourth-line setting.

### Safety profile

The safety profile of daratumumab by treatment regimen is presented in Table 5. In the overall safety population, serious TEAEs were reported in 29 (13.7%) patients, with the highest frequencies reported in patients receiving daratumumab monotherapy (22.7%), daratumumab + PI + IMiD ± dexamethasone (17.2%), and daratumumab + PI ± dexamethasone (15.8%). Pneumonia (*n* = 10 [4.7%]) was the only serious event reported in ≥ 5 patients overall. Adverse drug reactions were reported in 40 (18.9%) patients overall; events reported in ≥ 5% of patients included leukopenia (6.6%), neutropenia (5.7%), and thrombocytopenia (5.7%). The frequency of adverse drug reactions was highest for daratumumab + IMiDs ± dexamethasone (27.8%; most common were leukopenia and neutropenia [12.5% each]) and lowest for daratumumab + other therapies (9.1%; chills [9.1%]). Infusion-related reactions were reported in 19 of 132 (14.4%) evaluable patients overall and ranged from 9.5% for daratumumab + PIs + IMiDs ± dexamethasone to 27.8% for daratumumab monotherapy. Although 7 (6.3%) and 28 (25.0%) patients tested positive for HBV surface antigen and core antibodies at baseline, respectively, no HBV reactivation was reported in the collected data. Overall, 25 (11.8%) patients had died as of the data cutoff date, with equal numbers due to progressive disease (*n* = 8), adverse events (n = 8), or other reasons (cause of death not disclosed by family, *n* = 9; Table 5). Of the patients who died, 6 (2.8%) died ≤ 2 months after initiating a daratumumab-based regimen, 10 (4.7%) died 2–6 months after, and 9 (4.2%) died > 6 months after. One patient died due to an AE related to daratumumab treatment (acute onset chronic liver failure); this patient had tested negative for HBV surface antigen at baseline.

**Table 5:** Safety profile of daratumumab-based regimens

*n* (%)	Overall (*n *= 212)	Daratumumab monotherapy (*n *= 22)	Daratumumab + dexamethasone (*n *= 21)	Daratumumab + PI ± dexamethasone (*n *= 57)	Daratumumab + IMiD ± dexamethasone (*n *= 72)	Daratumumab + PI + IMiD ± dexamethasone (*n *= 29)	Daratumumab + other agents (*n *= 11)
Serious TEAEs	29 (13.7)	5 (22.7)	2 (9.5)	9 (15.8)	7 (9.7)	5 (17.2)	1 (9.1)
Events in ≥ 1% of patients^a^							
Pneumonia	10 (4.7)	1 (4.5)	1 (4.8)	3 (5.3)	1 (1.4)	3 (10.3)	1 (9.1)
COVID-19	4 (1.9)	2 (9.1)	0	1 (1.8)	1 (1.4)	0	0
Adverse drug reactions	40 (18.9)	5 (22.7)	2 (9.5)	8 (14.0)	20 (27.8)	4 (13.8)	1 (9.1)
Events in ≥ 1% of patients^a^							
Leukopenia	14 (6.6)	1 (4.5)	0	2 (3.5)	9 (12.5)	2 (6.9)	0
Neutropenia	12 (5.7)	1 (4.5)	0	1 (1.8)	9 (12.5)	1 (3.4)	0
Thrombocytopenia	12 (5.7)	1 (4.5)	1 (4.8)	3 (5.3)	7 (9.7)	0	0
Lymphopenia	10 (4.7)	1 (4.5)	0	1 (1.8)	6 (8.3)	2 (6.9)	0
Hypogammaglobulinemia	9 (4.2)	1 (4.5)	0	3 (5.3)	4 (5.6)	1 (3.4)	0
Anemia	8 (3.8)	1 (4.5)	1 (4.8)	2 (3.5)	4 (5.6)	0	0
Chills	7 (3.3)	2 (9.1)	0	3 (5.3)	0	1 (3.4)	1 (9.1)
Chest discomfort	4 (1.9)	1 (4.5)	0	0	2 (2.8)	1 (3.4)	0
Blood IgA decreased	3 (1.4)	1 (4.5)	0	1 (1.8)	1 (1.4)	0	0
Blood IgM decreased	3 (1.4)	1 (4.5)	0	1 (1.8)	1 (1.4)	0	0
Discontinuations due to TEAEs	25 (11.8)	6 (27.3)	2 (9.5)	4 (7.0)	9 (12.5)	3 (10.3)	1 (9.1)
Deaths	25 (11.8)	3 (13.6)	2 (9.5)	7 (12.3)	9 (12.5)	2 (6.9)	2 (18.2)
Primary cause of death							
Progressive disease	8 (3.8)	3 (13.6)	1 (4.8)	0	3 (4.2)	0	1 (9.1)
Adverse event	8 (3.8)	0	1 (4.8)	4 (7.0)	2 (2.8)	1 (3.4)	0
Other/Unknown^b^	9 (4.3)	0	0	3 (5.3)	4 (5.6)	1 (3.4)	1 (9.1)

*IgA *Immunoglobulin A, *IgM *Immunoglobulin M, *IMiD *Immunomodulatory drug, *PI *Proteasome inhibitor, *TEAE *Treatment-emergent adverse event

^a^Events occurring in the indicated proportion of patients within the overall patient cohort

^b^Family chose not to disclose reason or cause of death

Among patients with renal insufficiency/failure (*n* = 32), adverse drug reactions were reported for 2 (6.3%) patients, serious TEAEs were reported for 8 (25.0%) patients, and deaths were reported for 6 (18.8%) patients. Among patients aged ≥ 75 years (*n* = 21), adverse drug reactions were reported for 3 (14.3%), serious TEAEs were reported for 4 (19.0%) patients, and death was reported for 1 (4.8%) patient. As most patients were aged < 75 years (*n* = 191), the safety profile for this subgroup was similar to that of the overall study population.

## Discussion

This ongoing observational study provides insights into real-world physician treatment decisions, treatment patterns, and characteristics of Chinese patients with MM, in addition to clinical outcomes following daratumumab-based treatment. Daratumumab was most commonly administered in combination with a PI and/or IMiD ± dexamethasone. Response rates were high (ORR, 71.8%; ≥ VGPR, 51.4%) for patients who received daratumumab-based treatment, with higher rates observed for those who received daratumumab combination regimens. Furthermore, the safety profile of daratumumab-based regimens was generally consistent with the profile reported in interventional clinical trials, with no new safety concerns. These results provide insights into the characteristics and treatment patterns of Chinese patients with MM in clinical practice and support the effectiveness of daratumumab in this population. While results were generally comparable to other studies, including real-world studies conducted in other countries, there were also some key unique observations.

A comprehensive understanding of a patient’s profile is crucial for informed and effective treatment decisions. In the current study, Chinese patients with MM were diagnosed at a median age of 61 years, which is similar to that noted in Latin America (63 years) [18] but notably lower than reported in the United States (69 years) [19] and Europe (72 years) [20]. The distribution of MM incidence by sex was comparable (males: 57.5% [current study], 49.3%, 54%, and 58%, respectively). Notably, Chinese patients had a higher frequency of advanced disease, with 21.3%, 37.6%, and 41.1% of patients classified as ISS stage I/II/III respectively. This trend aligns with a study by the Asian Myeloma Network that reported rates of 19.9%, 36.1%, and 44.0%, respectively, in Chinese patients with MM [21], as well as real-world data for patients from Latin America (27.4%, 28.6%, and 44%) [18] and Europe (12%, 28%, and 60%) [20]. In contrast, a real-world study using data from the Flatiron Health database that reported a much lower frequency of advanced disease in US patients with MM, with rates of 18%, 18%, and 18% (46% unknown) [19]. The current study of Chinese patients with MM also found a high proportion of patients with high-risk cytogenetics (50% of evaluable patients). Comparison of high-risk cytogenetics between studies is difficult due to variations in the cytogenetic abnormalities evaluated. US data from the Flatiron Health database reported 36% of patients with high-risk cytogenetics using a definition similar to the current study except for the exclusion of gain(1q21) [19], while a European study reported 39% of patients with high-risk cytogenetics but excluded both gain(1q21) and amp(1q21) from their definition [20]. The frequencies of individual cytogenetic abnormalities in the current study were similar to those reported in the Asian Myeloma Network study in Asian patients [21]. It is important to note that treatment allocation may be guided by the presence of specific baseline characteristics. For instance, in Chinese clinical practice, patients with advanced disease (ISS stage III) commonly received daratumumab + other agents and daratumumab + PI ± dexamethasone, whereas those with ISS stage I disease commonly received daratumumab + IMiD ± dexamethasone.

Results from this analysis suggest treatment patterns for Chinese patients with MM are comparable to those in other countries. In the first-line setting, most patients received a PI-based or PI + IMiD–based regimen (42% and 35%, respectively). Comparably, in a nationwide real-world study of US data from the Flatiron Health database, PIs and IMiDs were also commonly used in early treatment lines, with the most common first-line treatments being PI + IMiD–based (53.4%) regimens followed by chemotherapy-based (13.4%), PI-based (12.8%), and IMiD-based (11.5%) regimens [22]. Despite advances in MM treatment, high rates of treatment discontinuation persist, often due to disease progression. In the current study, 24.5% of Chinese patients initiated daratumumab due to prior progression, aligning with findings on treatment discontinuations from a systematic review of 45 RCTs (22.6%) [23]. Consequently, there is an unmet need for new, innovative therapies in early treatment lines. However, the use of daratumumab-based regimens was relatively low (17%) in the first-line setting, with daratumumab instead more frequently reported in the second-line setting. In the current study, prior to receiving daratumumab, most patients were previously exposed to a PI (82.1%; primarily bortezomib [80.2%]) and/or an IMiD (58.0%; primarily lenalidomide [51.9%]) with or without dexamethasone. Despite this previous exposure a large proportion of patients received second-line daratumumab in combination with a PI (50.9%), IMiD (62.5%), or PI + IMiD (48.3%), suggesting that in Chinese clinical practice standard-of-care therapies are often prescribed again in subsequent treatment lines with the addition of daratumumab. This observation could be due to the reported synergistic effects of daratumumab and its potential in mitigating resistance to other drug classes [24]. The use of daratumumab monotherapy was more common among patients aged ≥ 75 years (23.8%) compared to the overall study population (10.4%), which is not unexpected given the frailer nature of this age subgroup. Notably, in the current study, 62.3% of patients initiated daratumumab-based regimens based on physician recommendation, primarily due to the observed deeper clinical responses in real-world settings.

In the current study, treatment responses were favorable in Chinese patients receiving daratumumab-based regimens, with an ORR and ≥ VGPR rate of 71.8% and 51.4%, respectively. Of note, the ORR remained high across most evaluated patient subgroups, although it was lower (57.9%) among patients aged ≥ 75 years, possibly due in part to the high use of daratumumab monotherapy and daratumumab + dexamethasone in older patients; however, the small size (*n* = 21) of this subgroup limits any clinically meaningful conclusions. Among the daratumumab-based regimens with the largest sample sizes, daratumumab + IMiD ± dexamethasone (*n* = 64) and daratumumab + PI ± dexamethasone (*n* = 45), ORR was achieved by 75.0% and 71.1%, respectively, and ≥ VGPR by 54.7% and 48.9%. These findings are consistent with a real-world analysis of MM patients in the United States, where daratumumab-based regimens yielded an ORR and ≥ VGPR rate of 71.3% and 47.3% [25], but are higher than the ORR of 55.9% and ≥ VGPR rate of 35.8% reported for daratumumab-based regimens in a recent real-world analysis of patients in Turkey [26]. One key consideration when making treatment decisions is the line of treatment in which therapies are initiated. In the current analysis, the median duration of exposure to daratumumab was longer in patients who initiated daratumumab in the first-line setting (9.7 months) compared to the second-line (6.2 months), third-line (5.8 months), and fourth-line (4.7 months) settings. These trends in effectiveness and duration of exposure further support the early use of daratumumab-based treatment for MM. Of note, while the follow-up in the current analysis is relatively short (10.5 months) for response and survival outcomes, this study is ongoing and more efficacy data will be presented within the next data cut.

The safety of treatment regimens also impacts treatment decision making in the real-world, and the importance of real-world safety evidence has increasingly become recognized because it allows for the evaluation of treatments beyond the tightly controlled settings and restricted patient populations often included in RCTs [27]. In the current study, serious TEAEs were reported in 29 (13.7%) patients overall, with the most commonly reported serious TEAE overall being pneumonia (4.7%). Adverse drug reactions were reported in 40 (18.9%) patients overall, the most common being leukopenia (6.6%), neutropenia (5.7%), and thrombocytopenia (5.7%). The occurrence of these types of adverse drug reactions are consistent with that observed in other previous clinical trials of daratumumab-containing regimens [8, 28]. Additionally, the safety profile of daratumumab-based regimens in patients with renal insufficiency/failure, as well as both younger (< 75 years) and older (≥ 75 years) patients, was consistent with the overall study population, with no unexpected findings. One common safety observation in Chinese patients with MM is a relatively high rate of infections. China has been shown to have a relatively high prevalence of HBV infection, although rates have progressively decreased over time. In a recent meta-analysis of 3740 studies, with over 231 million individuals from 6 Chinese regions, estimated HBV surface antigen seroprevalence for the general population decreased from 9.6% in 1973–1984 to 3.0% in 2021 [29]. In contrast, in the current study, 7 (6.3%) evaluable Chinese patients with MM tested positive for HBV surface antigen and 28 (25.0%) tested positive for HBV core antibodies at baseline, prevalences that are much higher than that observed in the general population. Even when taking into consideration the baseline age of pts at MM diagnosis in the current study (64 years), the rate of HBV infection remained higher in the current study than those aged ≥ 60 years in the general population (6.3% vs 4.6%) [29]. Despite the higher prevalence of HBV infection among Chinese patients, no patient who tested positive for HBV surface antigen or HBV core antibodies at baseline in the current study reported reactivation of HBV. Given that anti-CD38 monoclonal antibody treatments such as daratumumab have been associated with an increased risk of HBV reactivation [30], the lack of reactivation observed here is noteworthy. All in all, as per the current analysis, no unexpected or new safety concerns were observed. Given the relatively short follow-up time of this analysis, a more comprehensive comparison of safety outcomes will be explored in the next reported data cut.

Consistent with other real-world studies, one limitation of the current study is that the included data are restricted to information available within patient medical charts and electronic case report forms completed at each study site; thus, data may not be complete for all patients. Furthermore, data presented here from the first interim analysis of this study are from a relatively short median follow-up of 10.5 months, limiting the evaluation of longer-term outcomes such as survival and duration of response. However, this multicenter, noninterventional, observational study is ongoing and additional follow-up will provide further insights.

In conclusion, this retrospective, multicenter study is one of the largest and first of its kind to investigate patient characteristics, treatment patterns, and outcomes in Chinese patients with MM who were treated with daratumumab-based regimens in a real-world setting. The study revealed that Chinese patients with MM were diagnosed at a relatively young age and with a high prevalence of advanced disease and high-risk cytogenetics compared with patients in some other global regions. Daratumumab-based regimens were primarily utilized as a second-line treatment option, recommended by physicians due to their ability to produce deep and durable responses. The observed clinical outcomes were comparable to those reported in other real-world analyses and RCTs, with no new safety concerns. These findings support the use of daratumumab-based therapies as a standard of care for Chinese patients with MM patients. Future long-term data collection will provide additional insights into real-world treatment patterns and clinical benefits, potentially optimizing treatment guidelines and practices.

## Supplementary Information

Additional file 1. Table A. Summary of HBV results over time by daratumumab-based regimen.

Additional file 2. Figure A. Daratumumab treatment duration by line of therapy for daratumumab initiation.

Additional file 3. Figure B. Achievement of ≥VGPR (A) and ≥CR (B) by daratumumab-based regimen.

Additional file 4. Table B. Response to daratumumab by patient and treatment subgroups.

Additional file 5. Table C. Response to daratumumab overall and by daratumumab line of treatment.

Additional file 6. Table D. PFS and OS by daratumumab-based regimen.

## Data Availability

All relevant data for this analysis are included in the manuscript and supplemental files.

## Abbreviations

CI: Confidence interval
CR: Complete response
D-Pd: Daratumumab plus pomalidomide/dexamethasone
D-Rd: Daratumumab plus lenalidomide/dexamethasone
D-Vd: Daratumumab plus bortezomib/dexamethasone
D-VRd: Daratumumab plus bortezomib/lenalidomide/dexamethasone
ECOG: Eastern Cooperative Oncology Group
Ig: Immunoglobulin
IgA: Immunoglobulin A
IgGκ: Immunoglobulin G kappa
IgM: Immunoglobulin M
IMiD: Immunomodulatory drug
IMWG: International Myeloma Working Group
ISS: International Staging System
MM: Multiple myeloma
NDMM: Newly diagnosed multiple myeloma
NE: Not estimable
ORR: Overall response rate
OS: Overall survival
PFS: Progression-free survival
PI: Proteasome inhibitor
PR: Partial response
RCT: Randomized controlled trial
Rd: Lenalidomide/dexamethasone
R-ISS: Revised International Staging System
RRMM: Relapsed/refractory multiple myeloma
SD: Standard deviation
TEAE: Treatment-emergent adverse event
Vd: Bortezomib/dexamethasone
VenDd: Daratumumab plus venetoclax/dexamethasone
VGPR: Very good partial response
VMP: Bortezomib/melphalan/dexamethasone
