# Supplementary material for: Real-world analysis of treatment patterns, effectiveness, and safety of daratumumab-based regimens in Chinese patients with newly diagnosed or relapsed/refractory multiple myeloma
Source: BMC Cancer. 2025 May 7;25:836. doi: 10.1186/s12885-025-13925-3 (PMC12057279; doi:10.1186/s12885-025-13925-3)
Supplement: Supplementary file 1 — Additional file 1. Table A. Summary of HBV results over time by daratumumab-based regimen. [file 12885_2025_13925_MOESM1_ESM.docx]

**Additional file 1: Table A. Summary of HBV Results Over Time by Daratumumab-based Regimen**

|  | Overall  (n=212) | Daratumumab monotherapy  (n=22) | Daratumumab + dexamethasone  (n=21) | Daratumumab + PI ± dexamethasone (n=57) | Daratumumab + IMiD ± dexamethasone (n=72) | Daratumumab + PI + IMiD ± dexamethasone (n=29) | Daratumumab + other agents  (n=11) |
| --- | --- | --- | --- | --- | --- | --- | --- |
| Baseline |  |  |  |  |  |  |  |
| HBV test, n | 114 | 10 | 14 | 28 | 43 | 14 | 5 |
| HBV surface antibody, n (%) |  |  |  |  |  |  |  |
| n | 112 | 10 | 14 | 27 | 43 | 13 | 5 |
| Negative | 71 (63.4) | 5 (50.0) | 6 (42.9) | 16 (59.3) | 30 (69.8) | 9 (69.2) | 5 (100) |
| Positive | 41 (36.6) | 5 (50.0) | 8 (57.1) | 11 (40.7) | 13 (30.2) | 4 (30.8) | 0 |
| HBV surface antigen, n (%) |  |  |  |  |  |  |  |
| n | 112 | 10 | 14 | 27 | 43 | 13 | 5 |
| Negative | 105 (93.8) | 9 (90.0) | 14 (100) | 24 (88.9) | 42 (97.7) | 12 (92.3) | 4 (80.0) |
| Positive | 7 (6.3) | 1 (10.0) | 0 | 3 (11.1) | 1 (2.3) | 1 (7.7) | 1 (20.0) |
| HBV e antibody, n (%) |  |  |  |  |  |  |  |
| n | 112 | 10 | 14 | 27 | 43 | 13 | 5 |
| Negative | 99 (88.4) | 9 (90.0) | 13 (92.9) | 21 (77.8) | 41 (95.3) | 11 (84.6) | 4 (80.0) |
| Positive | 13 (11.6) | 1 (10.0) | 1 (7.1) | 6 (22.2) | 2 (4.7) | 2 (15.4) | 1 (20.0) |
| HBV e antigen, n (%) |  |  |  |  |  |  |  |
| n | 112 | 10 | 14 | 27 | 43 | 13 | 5 |
| Negative | 112 (100) | 10 (100) | 14 (100) | 27 (100) | 43 (100) | 13 (100) | 5 (100) |
| Positive | 0 | 0 | 0 | 0 | 0 | 0 | 0 |
| HBV core antibody, n (%) |  |  |  |  |  |  |  |
| n | 112 | 10 | 14 | 27 | 43 | 13 | 5 |
| Negative | 84 (75.0) | 6 (60.0) | 11 (78.6) | 18 (66.7) | 34 (79.1) | 11 (84.6) | 4 (80.0) |
| Positive | 28 (25.0) | 4 (40.0) | 3 (21.4) | 9 (33.3) | 9 (20.9) | 2 (15.4) | 1 (20.0) |
| HBV core DNA, n (%) |  |  |  |  |  |  |  |
| n | 31 | 3 | 1 | 7 | 11 | 8 | 1 |
| Negative | 30 (96.8) | 3 (100) | 1 (100) | 7 (100) | 11 (100) | 8 (100) | 0 |
| Positive | 1 (3.2) | 0 | 0 | 0 | 0 | 0 | 1 (100) |
| Post-baseline^a^ |  |  |  |  |  |  |  |
| HBV test, n | 56 | 4 | 7 | 15 | 20 | 7 | 3 |
| HBV surface antibody, n (%) |  |  |  |  |  |  |  |
| n | 54 | 3 | 7 | 14 | 20 | 7 | 3 |
| Negative | 33 (61.1) | 2 (66.7) | 3 (42.9) | 8 (57.1) | 15 (75.0) | 2 (28.6) | 3 (100) |
| Positive | 21 (38.9) | 1 (33.3) | 4 (57.1) | 6 (42.9) | 5 (25.0) | 5 (71.4) | 0 |
| HBV surface antigen, n (%) |  |  |  |  |  |  |  |
| n | 54 | 3 | 7 | 14 | 20 | 7 | 3 |
| Negative | 53 (98.1) | 3 (100) | 7 (100) | 13 (92.9) | 20 (100) | 7 (100) | 3 (100) |
| Positive | 1 (1.9) | 0 | 0 | 1 (7.1) | 0 | 0 | 0 |
| HBV e antibody, n (%) |  |  |  |  |  |  |  |
| n | 54 | 3 | 7 | 14 | 20 | 7 | 3 |
| Negative | 49 (90.7) | 3 (100) | 7 (100) | 11 (78.6) | 19 (95.0) | 6 (85.7) | 3 (100) |
| Positive | 5 (9.3) | 0 | 0 | 3 (21.4) | 1 (5.0) | 1 (14.3) | 0 |
| HBV e antigen, n (%) |  |  |  |  |  |  |  |
| n | 54 | 3 | 7 | 14 | 20 | 7 | 3 |
| Negative | 54 (100) | 3 (100) | 7 (100) | 14 (100) | 20 (100) | 7 (100) | 3 (100) |
| Positive | 0 | 0 | 0 | 0 | 0 | 0 | 0 |
| HBV core antibody, n (%) |  |  |  |  |  |  |  |
| n | 54 | 3 | 7 | 14 | 20 | 7 | 3 |
| Negative | 44 (81.5) | 3 (100) | 6 (85.7) | 9 (64.3) | 17 (85.0) | 6 (85.7) | 3 (100) |
| Positive | 10 (18.5) | 0 | 1 (14.3) | 5 (35.7) | 3 (15.0) | 1 (14.3) | 0 |
| HBV core DNA, n (%) |  |  |  |  |  |  |  |
| n | 20 | 1 | 3 | 7 | 8 | 1 | 0 |
| Negative | 20 (100) | 1 (100) | 3 (100) | 7 (100) | 8 (100) | 1 (100) | 0 |
| Positive | 0 | 0 | 0 | 0 | 0 | 0 | 0 |

HBV, hepatitis B virus; IMiD, immunomodulatory drug; PI, proteasome inhibitor.

^a^HBV test performed after daratumumab initiation. If a patient had multiple tests, the test result with the worst situation was taken.
