# Supplementary material for: Real-world analysis of treatment patterns, effectiveness, and safety of daratumumab-based regimens in Chinese patients with newly diagnosed or relapsed/refractory multiple myeloma
Source: BMC Cancer. 2025 May 7;25:836. doi: 10.1186/s12885-025-13925-3 (PMC12057279; doi:10.1186/s12885-025-13925-3)

**Additional file 2: Figure A. Daratumumab treatment duration by line of therapy for daratumumab initiation.**


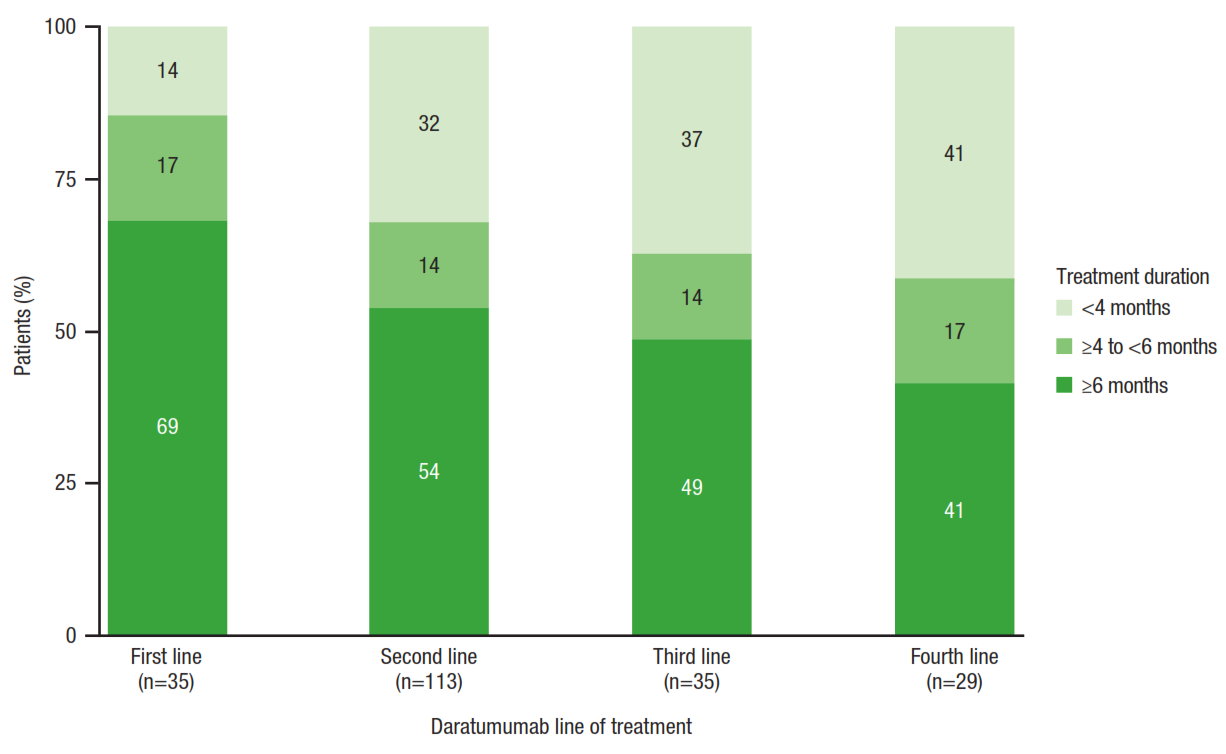

Supplement: Supplementary file 2 — Additional file 2. Figure A. Daratumumab treatment duration by line of therapy for daratumumab initiation. [file 12885_2025_13925_MOESM2_ESM.docx]
