# Supplementary material for: Real-world analysis of treatment patterns, effectiveness, and safety of daratumumab-based regimens in Chinese patients with newly diagnosed or relapsed/refractory multiple myeloma
Source: BMC Cancer. 2025 May 7;25:836. doi: 10.1186/s12885-025-13925-3 (PMC12057279; doi:10.1186/s12885-025-13925-3)
Supplement: Supplementary file 3 — Additional file 3. Figure B. Achievement of ≥VGPR (A) and ≥CR (B) by daratumumab-based regimen. [file 12885_2025_13925_MOESM3_ESM.docx]

**Additional file 3: Figure B. Achievement of ≥VGPR (A) and ≥CR (B) by daratumumab-based regimen.**

CR, complete response; IMiD, immunomodulatory drug; PI, proteasome inhibitor; VGPR, very good partial response.

^a^Cumulative combination of patients who achieved a true best response of VGPR, CR, or stringent CR for ≥VGPR in (A) and a true best response of CR or stringent CR for ≥CR in (B).

^b^Cumulative combination of patients who did not achieve a true best response of ≥VGPR or were not evaluable in (A) or ≥CR or not evaluable in (B).

**
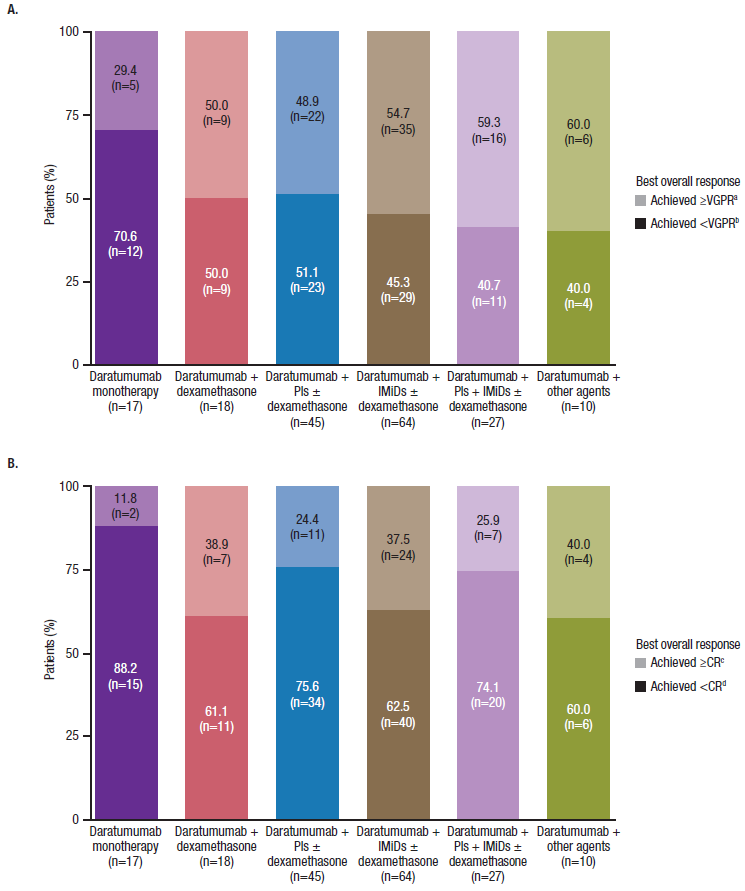
**
