# Supplementary material for: Real-world analysis of treatment patterns, effectiveness, and safety of daratumumab-based regimens in Chinese patients with newly diagnosed or relapsed/refractory multiple myeloma
Source: BMC Cancer. 2025 May 7;25:836. doi: 10.1186/s12885-025-13925-3 (PMC12057279; doi:10.1186/s12885-025-13925-3)
Supplement: Supplementary file 4 — Additional file 4. Table B. Response to daratumumab by patient and treatment subgroups. [file 12885_2025_13925_MOESM4_ESM.docx]

**Additional file 4: Table B. Response to Daratumumab in Patient and Treatment Subgroups**

|  | n | ORR,^a^ n (%) |
| --- | --- | --- |
| Renal insufficiency/failure^b^ | 28 | 22 (78.6) |
| Age |  |  |
| <75 years | 162 | 119 (73.5) |
| ≥75 years | 19 | 11 (57.9) |
| Cytogenetic risk^c^ |  |  |
| Standard risk | 32 | 25 (78.1) |
| High risk | 35 | 29 (82.9) |
| Last line of therapy before daratumumab initiation |  |  |
| Prior PI-based therapy | 59 | 43 (72.9) |
| Prior IMID-based therapy | 13 | 7 (53.9) |
| Prior PI + IMID–based therapy | 69 | 49 (71.0) |
| Daratumumab-based triplet regimen |  |  |
| Daratumumab + PI ± dexamethasone OR daratumumab + IMID ± dexamethasone | 109 | 80 (73.4) |
| Daratumumab + PI ± dexamethasone | 45 | 32 (71.1) |
| Daratumumab + bortezomib ± dexamethasone | 38 | 28 (73.7) |
| Daratumumab + ixazomib ± dexamethasone OR daratumumab + carfilzomib ± dexamethasone OR other regimen | 7 | 4 (57.1) |
| ASCT during or immediately after daratumumab-based treatment^d^ | 22 | 19 (86.4) |

ASCT, autologous stem cell transplant; IMID, immunomodulatory drug; ORR, overall response rate; PI, proteasome inhibitor.

^a^ORR was defined as partial response or better.

^b^Renal insufficiency/failure was assessed and indicated by the investigator.

^c^High cytogenetic risk was defined as ≥1 of the following high-risk cytogenetic abnormalities: t(4:14), t(14:16), del(17p), gain(1q21), amp(1q21), and/or t(14:20). Standard cytogenetic risk as defined as the absence of these high-risk cytogenetic abnormalities.

^d^ASCT received during the period of daratumumab treatment or immediately after daratumumab-based treatment and before initiation of a subsequent line of therapy.
