# Supplementary material for: Real-world analysis of treatment patterns, effectiveness, and safety of daratumumab-based regimens in Chinese patients with newly diagnosed or relapsed/refractory multiple myeloma
Source: BMC Cancer. 2025 May 7;25:836. doi: 10.1186/s12885-025-13925-3 (PMC12057279; doi:10.1186/s12885-025-13925-3)
Supplement: Supplementary file 5 — Additional file 5. Table C. Response to daratumumab overall and by daratumumab line of treatment. [file 12885_2025_13925_MOESM5_ESM.docx]

**Additional file 5: Table C. Response to Daratumumab Overall and by Daratumumab Line of Treatment**

| n (%) | Overall (n=181) | First line (n=29) | Second line (n=102) | Third line (n=27) | Fourth line (n=23) |
| --- | --- | --- | --- | --- | --- |
| ORR (≥PR) | 130 (71.8) | 23 (79.3) | 82 (80.4) | 13 (48.1) | 12 (52.2) |
| ≥VGPR | 93 (51.4) | 18 (62.1) | 63 (61.8) | 6 (22.2) | 6 (26.1) |
| Response category |  |  |  |  |  |
| Stringent CR | 5 (2.8) | 2 (6.9) | 3 (2.9) | 0 | 0 |
| CR | 50 (27.6) | 9 (31.0) | 34 (33.3) | 3 (11.1) | 4 (17.4) |
| VGPR | 38 (21.0) | 7 (24.1) | 26 (25.5) | 3 (11.1) | 2 (8.7) |
| PR | 37 (20.4) | 5 (17.2) | 19 (18.6) | 7 (25.9) | 6 (26.1) |
| Minimal response | 4 (2.2) | 0 | 2 (2.0) | 0 | 2 (8.7) |
| Stable disease | 18 (9.9) | 4 (13.8) | 4 (3.9) | 5 (18.5) | 5 (21.7) |
| Progressive disease | 13 (7.2) | 1 (3.4) | 6 (5.9) | 4 (14.8) | 2 (8.7) |
| Clinical relapse | 1 (0.6) | 0 | 1 (1.0) | 0 | 0 |
| Not evaluable | 14 (7.7) | 1 (3.4) | 7 (6.9) | 4 (14.8) | 2 (8.7) |

CR, complete response; ORR, overall response rate; PR, partial response; VGPR, very good partial response.
