# Supplementary material for: Real-world analysis of treatment patterns, effectiveness, and safety of daratumumab-based regimens in Chinese patients with newly diagnosed or relapsed/refractory multiple myeloma
Source: BMC Cancer. 2025 May 7;25:836. doi: 10.1186/s12885-025-13925-3 (PMC12057279; doi:10.1186/s12885-025-13925-3)
Supplement: Supplementary file 6 — Additional file 6. Table D. PFS and OS by daratumumab-based regimen. [file 12885_2025_13925_MOESM6_ESM.docx]

**Additional File 6: Table D. PFS and OS by Daratumumab-based Regimen**

|  | Overall  (n=212) | Daratumumab monotherapy  (n=22) | Daratumumab + dexamethasone  (n=21) | Daratumumab + PI ± dexamethasone (n=57) | Daratumumab + IMiD ± dexamethasone (n=72) | Daratumumab + PI + IMiD ± dexamethasone (n=29) | Daratumumab + other agents  (n=11) |
| --- | --- | --- | --- | --- | --- | --- | --- |
| PFS |  |  |  |  |  |  |  |
| Censored, n (%) | 163 (76.9) | 18 (81.8) | 17 (81.0) | 44 (77.2) | 52 (72.2) | 24 (82.8) | 8 (72.7) |
| Progression/deaths, n (%) | 49 (23.1) | 4 (18.2) | 4 (19.0) | 13 (22.8) | 20 (27.8) | 5 (17.2) | 3 (27.3) |
| Median (95% CI) PFS, mo | NE (19.3-NE) | NE (13.6-NE) | NE (9.9-NE) | NE (12.4-NE) | 19.4 (17.7-NE) | NE (13.2-NE) | NE (0.5-NE) |
| 6-month PFS, % | 84.3 | 83.9 | 88.9 | 82.8 | 84.5 | 86.1 | 80.0 |
| 12-month PFS, % | 75.0 | 83.9 | 74.1 | 71.6 | 73.6 | 86.1 | 68.6 |
| OS |  |  |  |  |  |  |  |
| Censored, n (%) | 187 (88.2) | 19 (86.4) | 19 (90.5) | 50 (87.7) | 63 (87.5) | 27 (93.1) | 9 (81.8) |
| Deaths, n (%) | 25 (11.8) | 3 (13.6) | 2 (9.5) | 7 (12.3) | 9 (12.5) | 2 (6.9) | 2 (18.2) |
| Median (95% CI) OS, mo | NE (NE-NE) | NE (NE-NE) | NE (NE-NE) | NE (NE-NE) | NE (17.7-NE) | NE (NE-NE) | NE (8.3-NE) |
| 6-month OS, % | 92.2 | 86.1 | 95.2 | 89.3 | 94.1 | 96.4 | 90.9 |
| 12-month OS, % | 88.9 | 86.1 | 87.3 | 86.8 | 90.5 | 96.4 | 81.8 |

CI, confidence interval; IMiD, immunomodulatory drug; NE, not estimable; OS, overall survival; PFS, progression-free survival; PI, proteasome inhibitor; PR, partial response.
